# Effect of Speed of Processing Training on Older Driver Screening Measures

**DOI:** 10.3389/fnagi.2017.00338

**Published:** 2017-10-17

**Authors:** Ranmalee Eramudugolla, Kim M. Kiely, Sidhant Chopra, Kaarin J. Anstey

**Affiliations:** Centre for Research on Ageing Health and Wellbeing, Australian National University, Canberra, ACT, Australia

**Keywords:** cognitive training, Useful Field of View, speed of processing, driving, older drivers, aging

## Abstract

**Objective:** Computerized training for cognitive enhancement is of great public interest, however, there is inconsistent evidence for the transfer of training gains to every day activity. Several large trials have focused on speed of processing (SOP) training with some promising findings for long-term effects on daily activity, but no immediate transfer to other cognitive tests. Here, we examine the transfer of SOP training gains to cognitive measures that are known predictors of driving safety in older adults.

**Methods:** Fifty-three adults aged 65–87 years who were current drivers participated in a two group non-randomized design with repeated measures and a no-contact matched control group. The Intervention group completed an average of 7.9 (*SD* = 3.0) hours of self-administered online SOP training at home. Control group was matched on age, gender and test-re-test interval. Measures included the Useful Field of View (UFOV) test, a Hazard Perception test, choice reaction time (Cars RT), Trail Making Test B, a Maze test, visual motion threshold, as well as road craft and road knowledge tests.

**Results:** Speed of processing training resulted in significant improvement in processing speed on the UFOV test relative to controls, with an average change of -45.8 ms (*SE* = 14.5), and effect size of ω^2^ = 0.21. Performance on the Maze test also improved, but significant slowing on the Hazard Perception test was observed after SOP training. Training effects on the UFOV task was associated with similar effects on the Cars RT, but not the Hazard Perception and Maze tests, suggesting transfer to some but not all driving related measures. There were no effects of training on any of the other measures examined.

**Conclusion:** Speed of processing training effects on the UFOV task can be achieved with self-administered, online training at home, with some transfer to other cognitive tests. However, differential effects of training may be observed for tasks requiring goal-directed search strategies rather than diffuse attention.

## Introduction

There is growing public interest and demand for computerized cognitive training programs that aim to enhance cognition and everyday function (Purcell and Rommelfanger, 2015). If such training leads to reliable improvement in cognitive functions that also translate to improvements in untrained daily activities, this could have significant impact on an aging population in terms of preventing functional decline and maintaining productivity. Computerized cognitive training typically focuses on a single cognitive domain or skill set, and uses repetition and an adaptive level of difficulty to achieve improvement in that skill. The efficacy of cognitive training has been the subject of multiple systematic reviews (Papp et al., 2009; Valenzuela and Sachdev, 2009; Martin et al., 2011; Kueider et al., 2012; Kelly et al., 2014). These indicate moderate improvement of performance on untrained but related cognitive tests when compared with controls receiving no contact (Valenzuela and Sachdev, 2009; Martin et al., 2011; Kueider et al., 2012). Few studies, however, measure ‘far transfer’ of training gains to everyday activities. Those that have examined this show inconsistent evidence for the generalizability of cognitive training effects (Papp et al., 2009; Martin et al., 2011; Casutt et al., 2014b; Kelly et al., 2014).

The ACTIVE trial represents one of the largest randomized controlled trials (*n* = 2802) of computerized cognitive training in older adults, with over 10 years of follow-up and examination of transfer to daily function (Jobe et al., 2001; Willis et al., 2006; Ball et al., 2013; Wolinsky et al., 2013; Rebok et al., 2014). In this trial, three groups were randomized to undergo 10, 1-h group-based sessions of computerized training on either memory, reasoning or speed of processing (SOP) over 5–6 weeks. SOP training was based on the useful field of view test (UFOV) (Ball and Owsley, 1993) in which participants view a computer display at increasingly brief exposure times during which they identify a central image on the screen while simultaneously noting the position of a peripheral object. Performance on the UFOV test is well established as a predictor of unsafe driving in older adults (Ball and Owsley, 1993; Ball et al., 2006). During SOP training, exposure times in the UFOV task are adaptively adjusted to maintain participant accuracy at 75%, with the aim of increasing speed of information processing through practice. The study found that compared to a no contact control group (*n* = 704), the SOP group (*n* = 712), demonstrated significant improvement on the UFOV test immediately after the training period, with no significant changes evident on cognitive tests of memory or reasoning (Ball et al., 2002). Importantly, the SOP group did not show change in a laboratory-based timed test of instrumental activities of daily living (IADLs) involving tasks like looking up a telephone number, finding food items on a crowded shelf, and responding to traffic signs (Ball et al., 2002). However, subsequent booster training at 1 year, and follow-up at 5–6 years post baseline demonstrated continued effects on the UFOV test, improvement in the timed IADLs (Willis et al., 2006), as well as a reduced rate of state-recorded at-fault crashes per mile relative to the control group (Ball et al., 2010). Other outcomes such as self-reported IADLs and driving habits remained unchanged.

Given the lack of far transfer immediately after training and the apparent delayed effect on functional outcomes 5–6 years post training, it is unclear what mechanisms underlie the observed changes in timed IADLs and reduced crash rate. Most activities of daily living involve multiple cognitive skills, are highly varied, and context dependent. Furthermore, performance based IADL measures do not correlate with informant or self-reported IADL difficulties, suggesting they may measure different constructs (Reuben et al., 1995; Owsley et al., 2002). Driving a car is one of the few complex IADLs that is relatively well defined in terms of scope and component tasks. Several speeded off-road measures have been developed that are context-specific to driving, are predictive of unsafe driving, and that tap into a range of driving-relevant cognitive skills (Reitan, 1985; Ball et al., 2006; Kay et al., 2009; Wetton et al., 2010; Unsworth et al., 2012). These include spatial attention (Reitan, 1985; Wetton et al., 2010), psychomotor response time and response inhibition (Wood et al., 2008), spatial planning (Kay et al., 2009; Unsworth et al., 2012), sequencing (Reitan, 1985) and visual motion perception (Wood et al., 2008). If the effects of SOP training on driving safety reflect a generalized increase in performance speed, then ‘near transfer’ may be apparent on other speeded tasks related to driving. Here, we examine the effects of SOP training, relative to no contact, on older adults’ performance on driving-specific measures of speed and attention as well as the UFOV test. We hypothesized that if SOP training gains transfer to other driving measures, then any effects seen on these measures would be associated with the magnitude and direction of effects seen on the UFOV test. We use self-administered online SOP training as this mode of intervention is the most translatable to real-world application.

## Materials and Methods

### Design

We used a two group non-randomized design with repeated measures and no contact, matched, control group. Individuals in the Intervention group were matched on age and test–retest interval to individuals in the Control group. The gender ratio was also matched between groups.

### Participants

The study recruited a total of 53 older adults (25 females) aged 65–87 [Mean = 71.91 years (*SD* = 4.47)] from the community. The Intervention group (*n* = 29) was recruited from the Canberra and surrounding regional New South Wales community (Australia) between December 2014 and February 2016. Four participants withdrew from the Intervention group due to time constraints (*n* = 1) and health-related reasons (*n* = 3). A matched participant could not be obtained for one participant in the Intervention group, leaving a final sample of *n* = 24 (10 females, Mean age = 71.3, *SD* = 3.9) in this group. The matched control participants (*n* = 24, Mean age = 71.9, *SD* = 4.3, 11 females) were recruited from the same community (*n* = 12) and also from community-dwelling older drivers already enrolled in a large ongoing observational study of older drivers (*n* = 12) between January 2015 and March 2016. Inclusion criteria for both groups were: current full driver’s license, aged over 65, and no current regular use of computerized brain training programs. Participation was voluntary and offered no reimbursement, apart from free access to the cognitive training software for the Intervention group. All participants provided written informed consent, and the protocol was approved by the ANU Human Research Ethics Committee.

### Measures

#### Demographics and Driving Habits Survey

All participants completed a questionnaire at the start of the study that collected data on age, gender, education level, falls history, physical activity levels, driving habits and experiences, and 12 month and 5-year accident history.

#### Cognitive Measures

Mini-Mental Status Exam: Global cognitive function was measured by the Mini Mental State Exam (Folstein et al., 1975).

*Trail Making Test B* (Reitan, 1985) is a test of cognitive flexibility and attentional shifting, which has been shown to be predictive of driving safety (e.g., Stutts et al., 1998). The participant is presented with a randomly distributed array of numbers (1–13) and letters (A–L) on a sheet of paper and is required to draw a line connecting the numbers and letters in ascending order, alternating between number and letter.

#### Driver Screening Measures

Useful Field of View^®^: This computer based test measures the threshold speed at which the participant can attend and process visual information on a screen with 75% accuracy (Sekuler and Ball, 1986). The task was presented on a Dell PC with a 17′′LCD display, with participants seated approximately 55 cm from the screen, and responses were input via the mouse. Only one subtest the UFOV^®^ was used. Here, participants attend to dual targets presented simultaneously on screen: a white, schematic image of a car or truck presented centrally with a second car figure presented randomly in one of eight locations along eight radial spokes (location from the upper vertical: 0°, 45°, 90°, 135°, 180°, 225°, 270°, or 315°) at a peripheral eccentricity of 10°. Following presentation, a random noise mask was shown and participants had to indicate: (a) which vehicle was presented in the center of the screen by pressing a picture of a car or truck; and (b) where the second car was located by pressing one of the eight possible locations onscreen. Display duration was varied between 17 and 500 ms using a double staircase procedure to estimate final threshold (Ball et al., 2006).

Hazard Perception Test: The ACT Hazard Perception test (Wetton et al., 2010) was presented on a Dell PC with 21.5-inch LCD touchscreen monitor. This is a video-based hazard perception test using clips filmed around the city of Canberra that mimic traffic situations where crashes are most likely to occur. The participant viewed each clip and pressed the relevant area of the touch-screen whenever they identify a potential hazard (traffic conflict). Twenty-two traffic conflicts (across 20 traffic clips of between 15 and 40 s duration) were presented. Performance was measured by calculating average standardized response times for all correctly identified hazards, and then expressed as a mean response time based on the mean and standard deviation of durations for all video clips. The response times were standardized to account for differences in clip duration.

OTDORA: Maze Test, and 14-Item Road Law and Road Craft Test. The Occupational Therapy-Drive Home Maze Test (OT-DHMT) is part of the OT-DORA Battery used in licensing recommendations for older and/or functionally impaired drivers (Krishnasamy and Unsworth, 2011; Unsworth et al., 2012). The Maze test in the OT-DORA battery requires the participant to trace a path through a drawn maze from the start to the finish, avoiding dead-ends or crossing maze boundaries. Time to completion was recorded. The Road Law and Road Craft test in the OT-DORA battery assesses the participant’s knowledge of road rules and comprises of a series of multiple choice and short answer questions as well as questions relating to diagrams of cars at an intersections.

DriveSafe/DriveAware Intersection Rules Test (Kay et al., 2008): The Intersections Rules subtest is part of the DriveSafe/DriveAware cognitive screening tool which is used to determine if older and cognitively impaired drivers require an on-road assessment. The subtest consists of eight diagrams of intersections with two to four cars in each intersection. The participant must use road marking and road signs presented to indicate the order in which the cars at the intersection should proceed.

Multi-D (Wood et al., 2008): This battery comprises three subtests assessing balance, sensitivity to visual motion and reaction time. An estimate of crash risk is calculated based on performance on the three subtests and self-reported average weekly kilometers driven.

Postural Sway Test: Postural sway (displacement of the body at the level of the waist) was measured with a sway meter with the participant standing on a foam rubber mat (40 cm × 40 cm × 15 cm thick) of medium density with their eyes closed.

Dot Motion Test: This computer-based test measured central motion sensitivity using random dot stimuli presented a working distance of 3.2 m. The test measures the threshold at which visual motion of randomly generated white dots on a black background can be detected. Within the total field of dots, a smaller section of dots that subtended at 2.9° and moved collectively in one of four directions (up, down, left, or right). Participants were asked to report the direction in which the dots moved. Thresholds were determined using a four alternative forced-choice staircase procedure.

Color Choice Reaction Task: This computer-based task measured foot and hand reaction time and response inhibition under forced-choice conditions. Participants viewed the image of either a blue or red car that was randomly presented in one of four quadrants of the screen, and were required to respond as rapidly as possible to the red car using either a manual button press (left or right corresponding to the left or right top quadrants of the screen, respectively) or a pedal press (left or right corresponding to left and right lower quadrants of the screen). The participant had to withhold a response if the car was blue.

### Intervention

The Intervention group received a gift subscription to Posit Science’s BrainHQ^®^ “Double Decision” cognitive training program, previously called “DriveSharp^®^.” This program is designed to improve participants’ useful field of view and visual processing speed (Ball et al., 1988; Wolinsky et al., 2013), and is based on the UFOV task. During training, speed of presentation and background complexity was adaptively reduced (between 17 and 500 ms) as the participant improved their performance. Participants in the Intervention group were instructed to complete 2-h per week of training over a period of 5 weeks and aim to accumulate a total of 10 h of training. Previous studies have reported effects on UFOV performance after an average of 8–10 h of self-administered training (Wadley et al., 2006; Ball et al., 2013; Wolinsky et al., 2013).

### Procedure

After providing written consent for involvement in the study, participants completed the demographic, health and driving questionnaire. All participants attended a baseline lab assessment where the cognitive and driver screening measures were administered. For participants in the Intervention group, a researcher then visited the participant’s home to set up access to the Double Decision task on their personal computer, provide instructions on its use and a training manual with training logging sheet and instructions. In addition, each participant in the Intervention group received a weekly phone call from the researcher to assist with technical issues, monitor motivation and adherence, and to provide support. The Control group received no contact from researchers during the period between baseline and follow-up testing. Follow-up testing involved a lab assessment comprising the same cognitive and driver screening measures as at baseline. Participants also completed a follow-up questionnaire on health and driving habits.

### Statistical Analysis

The effect of training on UFOV performance at follow-up was examined using Analysis of Covariance (ANCOVA) and regression, adjusting for baseline test performance and covariates including age, gender and time interval between assessments.

Transfer to other driver screening tests was examined using the same analysis approach for the DriveSafe Intersection, Multi-D, Trail Making Test B, Maze Test and the Hazard Perception test. Difference in rates of change in performance on these tests for the two groups was also examined using mixed models.

In order to examine the relationship between proximal effects of SOP training on the UFOV test and any training effects on the other driver screening tests (Test), *post hoc* analyses were conducted using generalized linear models of the change in each Test with the following predictors: group, gender, age, time interval between assessments, and the interaction between group and UFOV change scores. To adjust for potential influence of regression to the mean when using change scores, we included as covariates: the baseline UFOV score centered to the mean, and the baseline screening test score centered to the mean (Barnett et al., 2005).

## Results

### Baseline Characteristics

The Intervention and matched Control groups did not differ in level of education, MMSE, falls, driving distance, self-reported infringements or crashes (**Table 1**). The Intervention group reported slightly greater levels of moderate physical activity relative to the Control group, although this did not achieve significance (*p* = 0.067).

**Table 1:** Baseline sample characteristics.

	Control group (*n* = 24)		Cognitive training (*n* = 24)		*P*
Age, mean (*SD*)	71.9	(4.3)	71.3	(3.9)	0.599
Gender, *n* (%)					
Males	13	(54.2)	14	(58.3)	0.771
Females	11	(45.8)	10	(41.7)	
Education, *n* (%)					
Secondary	2	(8.3)	1	(4.2)	0.665
Technical	8	(33.3)	5	(20.8)	
Undergraduate	8	(33.3)	11	(45.8)	
Postgraduate	6	(25.0)	7	(29.2)	
MMSE, mean (*SD*)	29.0	(1.3)	29.4	(0.8)	0.180
Physical activity, mean (*SD*)					
Mild (hours per week)	7.9	(6.3)	8.8	(6.6)	0.640
Moderate (hours per week)	3.3	(3.5)	5.5	(4.5)	0.067
Vigorous (hours per week)	0.8	(1.3)	1.4	(2.3)	0.251
Number of falls in past 5 years^†^, *n* (%)					
None	19	(79.2)	21	(87.5)	0.439
One or more	5	(20.8)	3	(12.5)	
License class, *n* (%)					
Car	20	(83.3)	19	(79.2)	0.836
Medium rigid	1	(4.2)	2	(8.3)	
Heavy rigid	3	(12.5)	3	(12.5)	
Years driving, mean (*SD*)	51.5	(6.9)	53.0	(5.8)	0.423
Distance driven (km) per year^†^, *n* (%)					
<5000	4	(16.7)	4	(16.7)	0.441
5000 to 10000	9	(37.5)	12	(50.0)	
10000 to 15000	6	(25.0)	5	(20.8)	
15000 to 20000	4	(16.7)	3	(12.5)	
>20000	1	(4.2)	0	(0.0)	
Number of driving infringements in past year^†^, *n* (%)					
None	22	(91.7)	20	(83.3)	0.383
One or more	2	(8.3)	4	(16.7)	
Number of Accidents in past 5 years^†^, *n* (%)					
None	20	(83.3)	20	(83.3)	1.000
One or more	4	(16.7)	4	(16.7)	

*p-values calculated from χ^*2*^ for categorical variables and *t*-test for continuous variables. ^†^Self-reported*.

### Speed of Processing Training

Participants spent an average of 7.9 h (*SD* = 3.0, range: 2.5–12.7) over 16 sessions (*SD* = 8, range: 6–36) distributed over an average period of 43 days (*SD* = 17.4, range: 17–81) completing the UFOV training task. Barriers to completing the training included lack of time (*n* = 6), boredom (*n* = 8), eye strain (*n* = 5), and computer glitches (*n* = 6) or frustrations with the software (5 of those reporting ‘other’). Those who reported lack of time as a barrier completed an average of 3 fewer hours of training (*SE* = 1.3, *p* = 0.028). Those who reported boredom to be a barrier tended to complete 2.2 h more training (*SE* = 1.24, *p* = 0.09) than those who did not report boredom. One person reported a migraine and only completed 2.5 h of training but did not withdraw from the study.

### Effect of Speed of Processing Training on UFOV Performance

Compared to the control group, participants who completed UFOV training performed better at follow-up on the UFOV (**Table 2**). Cognitive training was associated with a decrease in SOP by 45.81 ms (*SE* = 14.47, *p* < 0.01), representing a moderate-sized (Kirk, 1996) effect (ω^2^ = 0.2) and explaining approximately 20% of the variance in UFOV scores at follow-up. When the model was further weighted for unequal selection to the Intervention group conditional on education and moderate physical activity, using doubly robust inverse probability weighted adjustment, the findings were similar (**Table 3**), with training being associated with a decrease of 35.8 ms (*SE* = 12.4, *p* < 0.01) relative to the control group.

**Table 2:** Regression analysis of effect of training on post-training UFOV test performance adjusted for baseline performance, age, gender, and time interval between test sessions.

	Cognitive training			
	*B*_1_	(*SE*)	*p*	effect size ω^2^
UFOV	-45.81	(14.47)	0.004	0.21
Cars RT	-0.08	(0.04)	0.034	0.07
Hazard perception	1.10	(0.33)	0.003	0.19
Maze test	-5.13	(1.70)	0.006	0.20
Trails B	-5.04	(6.14)	0.420	0.00
Dot motion test	-0.03	(0.03)	0.332	0.01
Drive safe intersections	0.46	(0.34)	0.186	0.03
Road law knowledge	-1.56	(0.76)	0.051	0.08

*Critical p-value is 0.017 correcting for the false discovery rate at 5% to adjust for eight multiple comparisons*.

**Table 3:** Average treatment effect from doubly robust inverse probability weighted regression adjustment.

	Cognitive training		
	β	(*SE*)	*p*
UFOV	-35.81	(12.41)	0.004
Cars RT	-0.06	(0.04)	0.123
Hazard perception	1.09	(0.31)	<0.001
Maze test	-3.59	(1.22)	0.003
Trails B	-3.52	(5.00)	0.482
Dot motion test 2	-0.02	(0.02)	0.248
Drive safe intersection	0.72	(0.32)	0.023
Road law knowledge	-1.29	(0.74)	0.080

*Critical p-value is 0.017 correcting for the false discovery rate at 5% to adjust for eight multiple comparisons*.

### Transfer of Training Effects to Other Driver Screening Batteries

Cognitive training was also associated with significant reduction in speed of completing the Maze test [-5.1 s (*SE* = 1.7), *p* < 0.01] and a significant increase in response times for detecting hazards in the Hazard Perception Test [1.10 s (*SE* = 0.33, *p* < 0.01] (**Table 4**). A non-significant trend for reduced reaction time was apparent in the Cars RT test [-0.08 (*SE* = 0.04), *p* = 0.03]. No changes were observed for road knowledge, intersection knowledge, and visual motion thresholds. The same pattern of findings was apparent after doubly robust inverse probability weighted adjustment (**Table 3**), although the estimates were more conservative for UFOV and the Maze test.

**Table 4:** Mean Pre and Post scores for each group on the driver screening measures.

		Control group				Intervention group			
		Mean	(*SD*)	Minimum	Maximum	Mean	(*SD*)	Minimum	Maximum
UFOV (ms)	Pre	90.0	(64.1)	17.0	197.0	108.8	(102.0)	17.0	373.0
	Post	78.2	(61.4)	17.0	214.0	29.0	(24.6)	17.0	127.0
Cars RT (ms)	Pre	0.92	(0.12)	0.8	1.3	0.91	(0.13)	0.7	1.2
	Post	0.91	(0.14)	0.7	1.3	0.83	(0.14)	0.6	1.1
Maze test (sec)	Pre	27.6	(7.3)	12.0	43.0	24.5	(14.0)	8.0	64.0
	Post	28.7	(8.4)	15.0	42.0	21.3	(9.3)	9.0	49.0
DriveSafe intersection test	Pre	6.7	(1.1)	4.0	8.0	6.5	(1.2)	4.0	8.0
	Post	6.2	(1.2)	3.0	8.0	6.7	(1.2)	4.0	8.0
Trail making test B (sec)	Pre	107.4	(36.1)	52.0	204.0	92.0	(48.6)	42.0	270.0
	Post	98.0	(43.9)	40.0	219.0	80.3	(34.1)	52.0	220.0
Hazard perception test (sec)	Pre	6.3	(1.4)	4.1	8.3	5.5	(1.1)	3.1	7.6
	Post	5.7	(1.3)	3.3	8.4	6.2	(1.4)	3.8	8.5
Dot motion test (log deg arc)	Pre	-1.87	(0.13)	-1.98	-1.51	-1.90	(0.13)	-2.0	-1.6
	Post	-1.89	(0.12)	-1.98	-1.51	-1.93	(0.07)	-2.0	-1.7
Road law knowledge	Pre	32.6	(3.4)	24.0	37.0	34.0	(2.6)	29.0	37.0
	Post	34.3	(2.3)	26.0	37.0	32.9	(2.3)	29.0	36.0

### Interactions between SOP Training, Change in UFOV Performance and Change in Other Driver Screening Batteries

**FIGURE 1:**
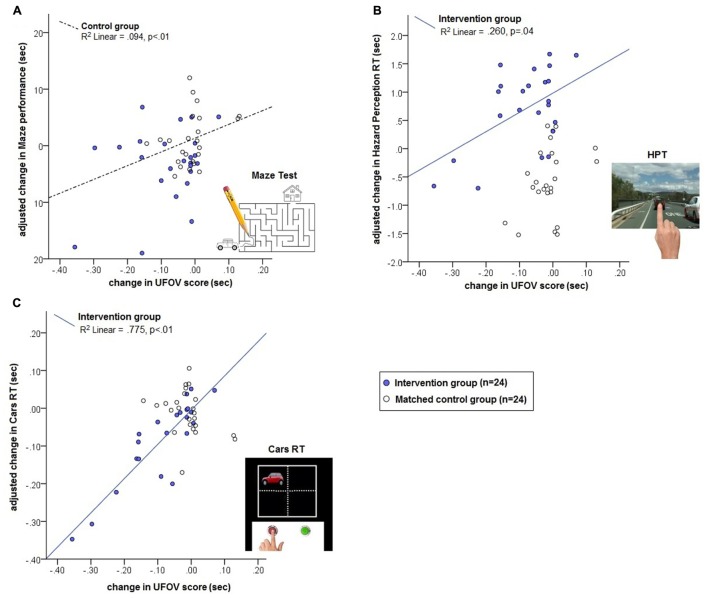
Model predicted change in performance on Maze test **(A)**, Hazard Perception test **(B)**, and Cars RT **(C)** as a function of change UFOV performance and group. Note: Maze Test image is representative and not the published test item.

Change in UFOV performance was computed as the difference between retest UFOV score and baseline UFOV score. Change scores for the Maze test, the Hazard Perception test and the Cars RT were similarly generated. To adjust for influence of regression to the mean, each baseline score for the above tests was centered to its mean. A generalized linear model with the change in Maze test scores was examined with group, gender, age, test–retest interval, centered baseline Maze test score, centered baseline UFOV score, and interaction between group × UFOV change. Results indicated a significant association between UFOV change and Maze test for the Control group [*B* = 0.032 (0.01), *p* = 0.008], but not the Intervention group [*B* = 0.006 (0.04), *p* > 0.10] (Supplementary Table 1). A model of change in HPT using the same predictors revealed a significant main effect of group [*B* = 1.60 (0.38), *p* < 0.001] and a marginal association between HPT change and UFOV change for the Intervention group [*B* = 0.009 (0.00), *p* = 0.036] and no association between HPT change and UFOV change for the control group [*B* = 0.003 (0.00), *p* > 0.10]. For the Cars RT test, there was a significant association between UFOV change and Cars RT change in the Intervention group [*B* = 0.001 (0.00), *p* = 0.003] but no such association in the control group [*B* = 0.00 (0.00), *p* > 0.10]. The model adjusted change scores for each driver screening measure was plotted as a function of change in UFOV score and SOP training group and is presented in **Figure 1** (see Supplementary Figure 1 for plots of unadjusted change scores).

### Association between SOP Training Intensity and Change in UFOV Performance

To examine whether training intensity, quality or length of time were most predictive of training gains in UFOV performance, a generalized linear model was conducted with change in UFOV as the dependent variable and the following predictors: number of hours of training, number of days of training, length of training period in days (i.e., including non-training days), and the test–retest interval. We further adjusted for age, centered baseline UFOV score, self-reported boredom as a barrier to training, and self-reported lack of time as a barrier to training. The results indicated that centered baseline UFOV [*B* = -1.04 (0.06), *p* < 0.001], length of training period in days [*B* = -1.05 (0.41), *p* = 0.011], and self-reported boredom [*B* = 21.31 (9.78), *p* = 0.029] were significant predictors of change in UFOV. Other measures of training intensity were not predictive of change in UFOV.

## Discussion

We found that relative to a no contact control group, older adults who engaged in self-administered online SOP training showed significant improvement in their SOP as measured by the UFOV test. SOP training was also associated with faster completion of a pen-and-paper Maze test, but slower detection of hazards when viewing video footage of urban driving. Trends toward faster responding on a test of foot and hand reaction time was also apparent following SOP but did not reach statistical significance.

The effect of SOP training on UFOV performance is consistent with numerous previous reports (Ball et al., 2002; Roenker et al., 2003; Wadley et al., 2006; Belchior et al., 2013; Wolinsky et al., 2013). We found that an average of 8 h of training was associated with an average reduction in processing time of 45.8 ms (*SE* = 14.47), which is within the range of effects reported in other small studies of SOP training in older adults (Roenker et al., 2003; Edwards et al., 2006; Belchior et al., 2013), and one trial that included middle aged adults (50–64 years) (Wolinsky et al., 2013). In our study, 96% of participants demonstrated improvement in UFOV performance following SOP training, whereas 63% demonstrated improvement without training. It is also notable that while 13% of the sample achieved ceiling performance on the UFOV at baseline, this was 54% following SOP training and 4% after no contact. Baseline UFOV performance in some previous studies are significantly lower than that in our study, suggesting that many older adults are already at ceiling on this outcome measure. It is plausible that those close to ceiling on UFOV may demonstrate smaller gains from SOP training, however, reports from the ACTIVE trial suggest training gains are unrelated to factors such as age (Ball et al., 2013), which is otherwise related to performance on the UFOV (Edwards et al., 2006). Nevertheless, our study demonstrated SOP training effects with proximal transfer to the primary outcome measure.

We did not find a significant improvement in a foot and hand choice reaction time task following SOP training. This contrasts with (Roenker et al., 2003) who reported that SOP training was associated with an improvement relative to baseline on a choice reaction task involving a left or right steering response to target road signs. This study, however, lacked a comparable control group and although participants demonstrated an improvement of 0.86 SD units from baseline, their post-training performance remained slower than that of a low-risk no contact reference group. In our study, the Intervention group demonstrated an improvement of 0.61 SD units relative to baseline on the Cars RT task, compared to a change of 0.08 SDs in the matched control group – although this difference did not reach our threshold for statistical significance. It is possible that with greater statistical power, a transfer of training gains to foot and hand choice reaction times would be apparent. Although the effect of SOP training did not reach significance, there was a significant association between the degree of change in UFOV performance and the change in Cars RT performance in the SOP training group (**Figure 1**). This relationship suggests that individuals who experienced enhanced processing speed in the UFOV task following training, experienced a similar enhancement of foot and hand reaction times to unpredictable visual stimuli in the Cars RT test. In contrast, such a relationship was not evident for the no contact control group. This relationship further supports the idea that a transfer of training gains may be observed with larger samples.

Although the Hazard Perception test is similar to the UFOV and Cars RT in that it is computerized and requires rapid perception of brief or dynamic visual stimuli, the hazard stimuli are not entirely unpredictable. The test is designed to reflect real-world perception of potential traffic hazards, relying on the observer’s knowledge of common hazards, their location and anticipation of movement trajectories (Wetton et al., 2010). Our results indicated that SOP training was associated with a significant slowing in participants’ detection and response to traffic hazards. This main effect of group was confirmed when the relationship between UFOV change and HPT change was examined. Although the Intervention group demonstrated a change in intercept, there was a linear trend suggesting that those who showed increased speed on the UFOV tended to have either a smaller degree of slowing or slightly faster responding in the HPT (**Figure 1**). The effects cannot be explained by a speed-accuracy trade-off as accuracy on the HPT was already near ceiling. One possibility is that the HPT requires a goal directed search strategy, whereas SOP training targets automatic or stimulus-driven capture of attention by spreading the attentional focus in a diffuse manner across the whole visual field (Ball and Owsley, 1993; Ball et al., 2013). If the Intervention group used this strategy of allowing stimuli to capture attention, they may have been at a disadvantage compared to targeting their attention in a top-down manner to anticipate potential hazards. Previous studies on search strategy and fixation patterns in hazard perception suggest experienced drivers prioritize attention to specific regions of the visual field, particularly the horizontal meridian, but adapt their horizontal visual search strategy to the type of road and traffic condition, whereas inexperienced drivers spread fixations equally (Chapman and Underwood, 1998; Crundall, 2005; Falkmer and Gregersen, 2005). Further studies are required to examine the relationship between SOP training and possible differential effects on activities that require top-down as opposed to stimulus-driven attention. Studies of training effects on defensive observation skills during real or simulated driving is also warranted. There is some evidence that repetition or training on a specific task can lead to maladaptive effects on other tasks (May, 2011), and is sometimes referred to as ‘negative plasticity’ (Mowszowski et al., 2010). However, the observed effects on their own are unlikely to represent negative plasticity as there was a positive relationship between improved speed on the UFOV and improved speed on the HPT. In the context of both this and the similar positive relationship with change in Cars RT speed, it is possible that training led to improved speed of stimulus-driven attention and psychomotor response, but not goal directed visual search. Future work will need to examine this hypothesis and the effect of UFOV training on visual search strategy and eye movements.

We did not find any transfer of SOP training effects to the Trail Making Test B. To our knowledge, no studies have examined the immediate transfer of SOP training gains to neuropsychological tests such as the Trail Making Test B, however, Wolinsky et al. (2013) report significant improvement in several speeded cognitive tests, including Trail Making Test B, one year following home-based SOP training. In the absence of both immediate and long-term follow-up data on the one sample, it is not possible to understand whether effects potentiate over time. In contrast to Trails B, our findings suggest an immediate effect for the Maze Test, another paper and pencil speeded cognitive task. Although the observed effect size was similar to that for the UFOV test, the Intervention group did not show an association between change in UFOV scores and Maze scores, whereas such a relationship was apparent in the control group. The lack of random allocation and an active control in the present study means that it is unclear what is underlying this interaction. One possibility is that participants receiving the intervention may also have been more motivated to perform better on the Maze test, particularly given a lack of relationship to changes in UFOV speed.

Our data suggested that training gains on the UFOV task were better predicted by the overall length of time over which training occurs, rather than the hours spent directly in training. Although this was based on a small sample, and other factors such as boredom influenced training participation and outcome. Previous data from other forms of computerized cognitive training such as working memory training suggest that distributed training rather than high intensity training is more beneficial (Penner et al., 2012). Future studies may benefit from using training protocols that emphasize motivation, rest periods between training sessions and training over a longer period of around 2 months.

While the present study was focused on the transfer of gains from computerized SOP training to other cognitive measures associated with on-road driving safety, there are other modes of computer-based training shown to improve cognitive performance (Casutt et al., 2014a, 2016) as well as on-road driving performance (Casutt et al., 2014b). The latter study reported that compared to older adults who engaged in attention and vigilance training in a driving simulator, older adults who received driving-specific training in a simulator showed subsequent improvement in on-road driving behaviors (Casutt et al., 2014b). Training on the driving simulator involved ten 40 min sessions over 5-weeks, where drivers traveled in simulated routes of increasing complexity, receiving verbal feedback on their speed of reactions and driving errors after each session (i.e., accidents, road rule violation, lane positioning, reaction time to hazards). Thus, it is possible that complex interventions targeting both cognitive and behavioral aspects of driving may translate more effectively to on-road performance.

In summary, our study shows that self-administered SOP training is feasible and improves performance on the UFOV test. Our findings suggest immediate effects on other cognitive tests known predictors of driving safety – however, we postulate that while some changes may reflect transfer of training gains in processing speed, others may be driven by changes in strategy. Significant limitations of the study include lack of an active control group and non-randomized allocation to groups, so the findings need to be interpreted with caution. There is evidence that SOP training is associated with gains in self-reported every day functioning (Ball et al., 2002) and also self-rated cognitive improvement, sometimes in the absence of objective improvement (Bray et al., 2016). Further work is required to examine training effects in the context of activities relying on different attentional strategies, changes in self-efficacy, and expectations of aging.

## Author Contributions

KA, RE, and KK were responsible for study concept and design. KK and RE conducted data analysis. SC conducted data collection and participant matching. All authors contributed to interpretation, manuscript write-up and revision.

## Conflict of Interest Statement

The authors declare that the research was conducted in the absence of any commercial or financial relationships that could be construed as a potential conflict of interest.
