# Supplementary material for: Effect of Speed of Processing Training on Older Driver Screening Measures
Source: Front Aging Neurosci. 2017 Oct 17;9:338. doi: 10.3389/fnagi.2017.00338 (PMC5651014; doi:10.3389/fnagi.2017.00338)
Supplement: Supplementary file 1 [file Table_1.DOCX]

**Supplementary Table 1.** Association between change in UFOV performance and change in other outcome measures.

|  |  | Parameter Estimate | |  |
| --- | --- | --- | --- | --- |
|  |  | B | (SE) | *P* |
| Maze Test change | Group (ref 2) | -4.66 | 1.80 | .01 |
|  | Sex (ref. female) | -2.92 | 1.52 | .06 |
|  | Age | .88 | .21 | <.001 |
|  | Pre-Post Interval | -.08 | .04 | .02 |
|  | Group 1 x UFOV change | .01 | .02 | .73 |
|  | Group 2 x UFOV change | .03 | .01 | .01 |
|  | Centred baseline Maze | -.45 | .07 | <.001 |
|  | Centred baseline UFOV | -.00 | .02 | .95 |
|  |  |  |  |  |
| Hazard Perception change | Group (ref 2) | 1.60 | .38 | <.001 |
|  | Sex (ref. female) | -.40 | .36 | .27 |
|  | Age | .00 | .07 | .95 |
|  | Pre-Post Interval | -.01 | .00 | .49 |
|  | Group 1 x UFOV change | .01 | .00 | .04 |
|  | Group 2 x UFOV change | .00 | .01 | .61 |
|  | Centred baseline HPT | -.40 | .14 | .00 |
|  | Centred baseline UFOV | .01 | .01 | .31 |
|  |  |  |  |  |
| Cars RT change | Group (ref 2) | .01 | .03 | .80 |
|  | Sex (ref. female) | .01 | .04 | .80 |
|  | Age | .01 | .00 | .38 |
|  | Pre-Post Interval | -.00 | .00 | .25 |
|  | Group 1 x UFOV change | .00 | .00 | <.01 |
|  | Group 2 x UFOV change | .00 | .00 | .93 |
|  | Centred baseline Cars RT | -.43 | .18 | .02 |
|  | Centred baseline UFOV | .00 | .00 | .33 |
|  |  |  |  |  |

*Note:* Models use robust estimation. Reference for Group is controls, reference for Sex is females. Group 1 is Intervention, Group 2 Controls. Change scores are difference between baseline and follow-up test scores. All dependant variable units are in seconds and UFOV change is in milliseconds.

**Supplementary Figure Caption.** Unadjusted change in performance on the Maze test, HPT and Cars RT tests as a function of UFOV change and group.
