# Supplementary figures and images for: Effect of Speed of Processing Training on Older Driver Screening Measures
Source: Front Aging Neurosci. 2017 Oct 17;9:338. doi: 10.3389/fnagi.2017.00338 (PMC5651014; doi:10.3389/fnagi.2017.00338)

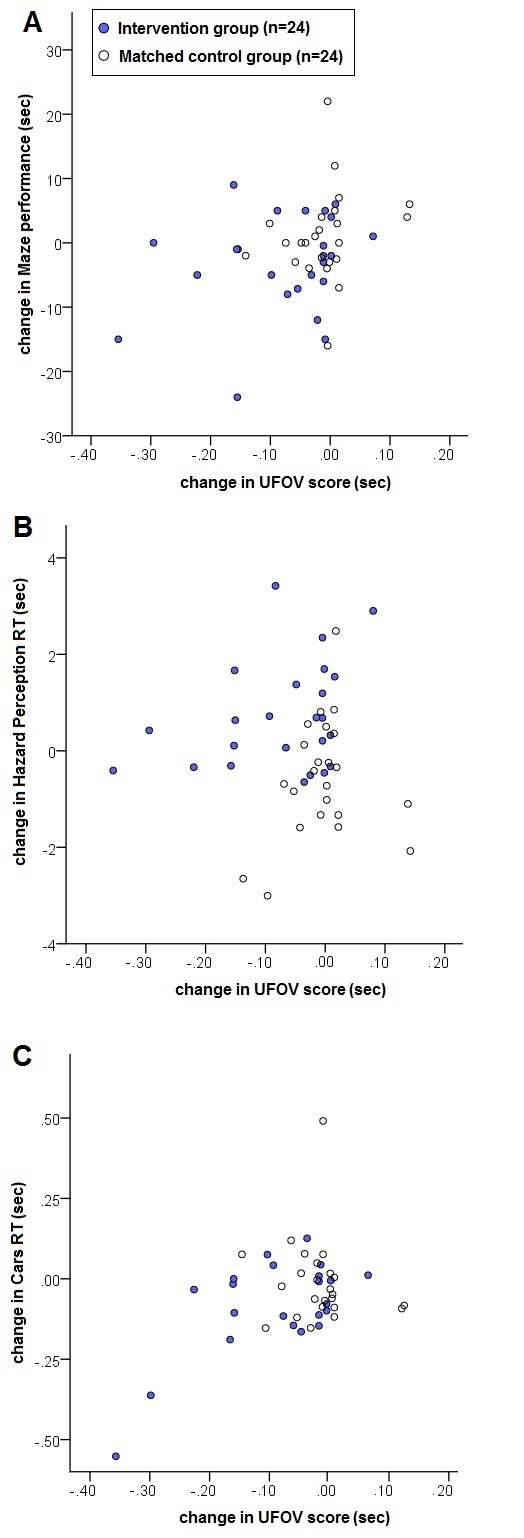

Supplement: Supplementary file 2 [file Image_1.JPEG]
